# Rectal cancer staging using MRI: adherence in reporting to evidence-based practice

**DOI:** 10.1177/0284185120906663

**Published:** 2020-02-27

**Authors:** Gustav Alvfeldt, Peter Aspelin, Lennart Blomqvist, Nina Sellberg

**Affiliations:** 1 Department of Learning, Informatics, Management and Ethics, Karolinska Institutet, Stockholm, Sweden; 2 Department of Clinical Science, Intervention and Technology, Karolinska Institutet, Stockholm, Sweden; 3 Department of Molecular Medicine and Surgery, Karolinska Institutet, Stockholm, Sweden; 4 Department of Imaging and Physiology, Karolinska University Hospital, Stockholm, Sweden

**Keywords:** Magnetic resonance imaging, rectum, staging, technical aspects, health policy and practice, computer applications–general

## Abstract

**Background:**

Magnetic resonance imaging (MRI) is the first-line imaging modality for local staging of rectal cancer. The radiology report should deliver all relevant available imaging information to guide treatment.

**Purpose:**

To explore and describe if there was a gap between the contents in MRI reports for primary staging of rectal cancer in Sweden in 2010 compared to evidence-based practice.

**Material and Methods:**

A total of 243 primary MRI staging reports from 2010, collected from 10 hospitals in four healthcare regions in Sweden, were analyzed using content analysis with a deductive thematic coding scheme based on evidence-based practice. Focus was on: (i) most frequently reported findings; (ii) correlation to key prognostic findings; and (iii) identifying if any findings being reported were beyond the information defined in evidence-based practice.

**Results:**

Most frequently reported findings were spread through the bowel wall or not, local lymph node description, tumor length, and distance of tumor from anal verge. These items accounted for 35% of the reporting content. Of all reported content, 86% correlated with the evidence-based practice. However, these included more information than was generally found in the reports. When adjusting for omitted information, 48% of the reported content were accounted for. Of the reported content, 20% correlated to key pathological prognostic findings. Six types of findings were reported beyond the evidence-based practice, representing 14% of the total reporting content.

**Conclusion:**

There was a gap between everyday practice and evidence-based practice in 2010. This indicates a need for national harmonization and implementation of standardized structured reporting templates.

## Introduction

Colorectal cancer is the third most common form of cancer in Sweden, affecting around 6200 people. Clinical staging of rectal cancer is performed to individualize treatment for best possible outcome (1,2). Magnetic resonance imaging (MRI) is considered the first-line imaging modality for the staging of rectal cancer. Several studies have been able to confirm the importance of using MRI to accurately stage the disease and the key prognostic elements when interpreting and reporting findings (1,3–5). According to the expert consensus panel of the European Society of Gastrointestinal and Abdominal Radiology, staging of rectal cancer should be “reported in a structured fashion so that important findings impacting directly on therapeutic decision making are not omitted” (5, p. 2523).

The results of the MERCURY study group, published during the mid-2000s (1,3,6) and describing the interpretation of images and acquisition techniques for MRI examinations of rectal cancer patients with particular focus on key pathological prognostic factors of importance, was followed by a number of publications in the field (4,7–10). This has contributed to a more standardized and systematic approach to staging, reading, and reporting of rectal cancer as well as clinical recommendations and practice guidelines (5,11).

Based on the variables and measurements described in the MERCURY studies and the subsequent articles, the Swedish Colorectal Cancer Registry (SCRCR) has developed radiology proforma for the reporting of rectal cancer patients to the registry (12). In 2014, the Swedish Society of Radiology (SFMR) released its first radiology reporting template for the primary staging of rectal cancer (13) based on the SCRCR proforma.

The aim of this empirical retrospective case study was to explore contents in Swedish free-text reports for the primary staging of rectal cancer using MRI authored in 2010. The aim was to identify how well the content in these radiology reports related to evidence-based practice (EBP) for the staging of rectal cancer in the form of Swedish national proforma (12,13). Particular focus was given to: (i) most frequently reported findings in reports; (ii) correlation to key pathological prognostic findings as described by the MERCURY study group; and (iii) identifying if any findings being reported were beyond the information found in the EBP.

## Material and Methods

### Sample size and filtering of radiology reports

The units of analysis in this study were reports on the primary staging of rectal cancer authored in 2010, collected from 10 hospitals in four healthcare regions in Sweden, the three largest regions and one smaller region (Table 1). The study protocol was vetted and approved by the Ethical Review Board. Patients reports were identified via the SCRCR and assorted by hospital and radiology department. The healthcare regions and the hospitals were chosen based on the number of rectal cancer patients at each site. Regions and hospitals with a high number of patients reported in the SCRCR were asked to contribute with data. Each radiology department provided de-identified reports from their Radiology Information System (RIS), filtered by the first pelvic MRI after diagnosis before treatment.

**Table 1.** Statistical overview of the reports at each hospital.

Healthcare region	Hospital	Compliant reports	Selected reports
Region Stockholm	Karolinska University Hospital	30	25
	Södersjukhuset	56	25
	Ersta Hospital	66	25
Region Skåne	Skåne University Hospital	72	25
	Kristianstad Hospital	18	18
	Helsingborg Hospital	37	25
Region Västra Götaland	Sahlgrenska University Hospital	88	25
	Södra Älvsborg Hospital	31	25
	Skaraborg Hospital	28	25
Region Uppsala	Uppsala University Hospital	39	25
	Total	467	243

A total of 730 reports were obtained. Due to issues with none-standardized RIS systems, none of the healthcare regions could filter out only the reports that were of interest to this study. Thus, several exclusion criteria needed to be applied manually on the given reports (Table 2). After applying the listed exclusion criteria, 467 reports were found to be compliant to this study (Table 1).

**Table 2.** Exclusion criteria for MRI reports.

No.	Exclusion criteria	Comment
1	Demonstration and/or multidisciplinary conference reports	These reports already have a previous “original” staging report
2	Reports written in any other year than 2010	This could happen since year of diagnoses does not always equal year of exam
3	Reports written by sub-contractors and not the actual hospital radiology department	I.e. referrals that were forwarded to, and answered by, another radiology department
4	Reports where the examined body part is something else than the abdominal region (abdomen, lower abdomen, pelvis, rectum)	Combinatory procedures are included if they combine abdominal MRI with some other procedure, e.g. liver CT for review of metastases
5	Reports where the modality is something other than a pelvic MRI	E.g. if the procedure is an abdominal CT
6	Non-staging exams	
7	Follow-up exams	E.g. a staging exam after neoadjuvant treatment
8	Cancelled exams	Such exams typically end up with a final report stating that the exam was cancelled in the observations or findings section
9	Exams without rectal tumor findings	E.g. if the reason for referral states a rectal cancer staging but the radiology findings does not concur there is a tumor to stage
10	Exams where the “reason for study” equals “status post op” or “relapse”	
11	Other kind of tumor findings	E.g. anal/colon cancer and not rectal cancer
12	Duplicates	Some datasets contained duplicates of reports

CT, computed tomography; MRI, magnetic resonance imaging.

The datasets were staged using Microsoft Excel version 2016 before imported in QSR International Nvivo 11 for coding and analysis. The staging phase involved steps and measures to harmonize the datasets from the different hospitals. The 467 compliant reports were randomized in Microsoft Excel using the RAND-function. Twenty-five randomized reports from each hospital were imported into Nvivo. One hospital ended up with a dataset of 18 reports, giving us a total amount of 243 reports, adequate to reach data saturation and information power (14,15) to make valid inferences about the content in communicated primary staging reports on rectal cancer.

### Annotating MRI reports using content analysis

The radiology reports were interpreted and coded using a deductive content analysis research technique by means of coding the report contents to pre-defined categories in a coding scheme with a thematic approach, i.e. dividing the content of the reports into shorter units or segments with shared thematic meaning (16–20).

**Fig. 1.**
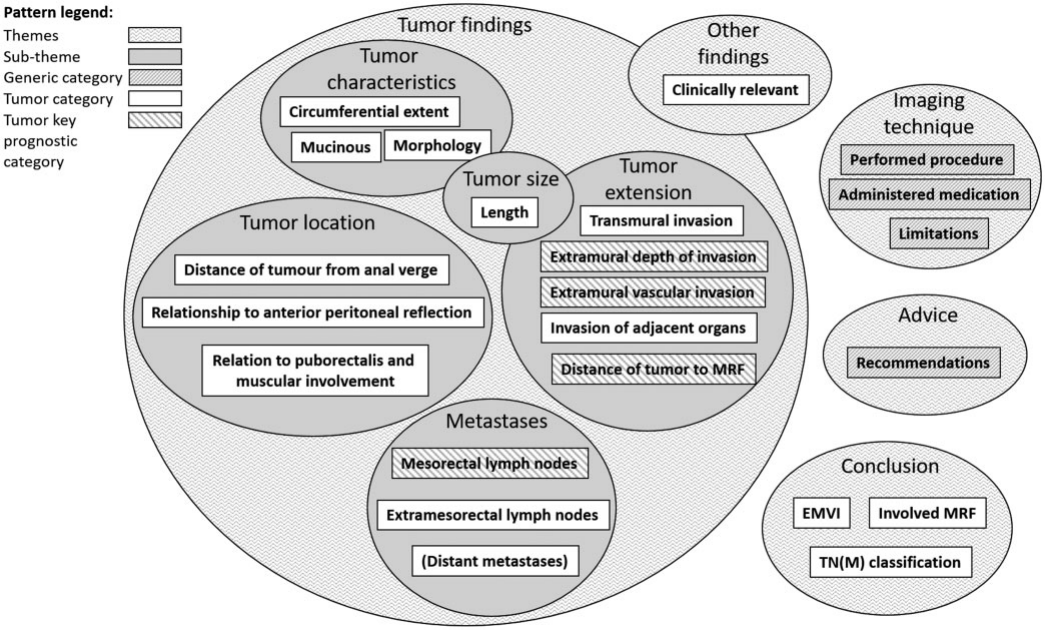
Visualization of pre-defined coding scheme.

The pre-defined coding scheme was created, consisting of: (i) themes; (ii) sub-themes; and (iii) categories. The themes and categories were based on EBP in the form of generic radiology practice guidelines (21,22) and the EBP behind the SCRCR proforma protocol and the SFMR national reporting template for the primary staging of rectal cancer (12,13). A visualization of the pre-defined coding scheme is shown (Fig. 1). A detailed description of the pre-defined coding scheme preparation process can be found in Appendix 1.

The unit of text to be coded, the recording unit, can be coded to the same theme and category if it shares the same semantics (17,18,23). Examples of coded recording units are presented in Tables 3 and 4. Recording units that could not be coded to any of the pre-defined categories were coded to a temporary category and later analyzed by an abdominal radiologist expert to determine if the they belonged to an existing category or a new category. In this way, new categories can be introduced like in an inductive approach (17,24,25).

**Table 3.** Examples of recording units and how they are coded to a category within a theme.

No.	Recording unit	Category	Sub-theme	Theme
1	The distance [of the tumor] to the MRF is short but is estimated to be >1 mm	Distance of tumor to MRF	Tumor extension	Tumor findings
2	No overgrowth on the bladder or vesicles. At the top, there are some intestines adjacent to the thickened rectal wall, hard to say if there is any overgrowth	Tumor invasion of adjacent organs		
3	[A tumor] that takes up large parts of the circumference	Tumor morphology	Tumor characteristics	
4	The lowest part of the tumor is approximately 3 cm above the anus	Distance of tumor from anal verge	Tumor location	
5	There is a suspected rectal cancer some decimeter up [from the anus]			
6	[Tumor is located] approximately 15 cm cranial of the external sphincter			
7	Paramedian on the left side corresponding to the tumor, shows pathological soft tissue dorsally of the prostate	Clinically relevant	n/a	Other findings
8	Left-sided hip prosthesis creates moderate artifacts on diffusion-weighted series	Limitations	n/a	Imaging technique

Content within square brackets are contextual notes written by the authors to facilitate reading of recording units that have been parted from longer sentences.

MRF, mesorectal fascia.

**Table 4.** Examples of sentences that have been divided into shorter units or segments and coded to different types of categories.

Sentences with multiple recording units				
“The polypous tumor without apparent mucinous element grows into the mesorectal fat with a 4-mm outlet that reaches 25 mm from the mesorectal fascia”				
No.	Recording unit	Category	Sub-theme	Theme
1	The polypous tumor	Morphology	Tumor characteristics	Tumor findings
2	[The tumor is] without apparent mucinous element	Mucinous		
3	[The tumor] grows into the mesorectal fat	Transmural invasion	Tumor extension	
4	[Tumor grows outside the rectal wall] with a 4-mm tumor outlet	Depth of invasion		
5	[A tumor outlet] that reaches 25 mm from the MRF	Distance of tumor to MRF		

MRF, mesorectal fascia.

By applying this approach on the total cohort of reports, all the recording units of the reporting content have been allocated to a specific theme and category, pre-defined or new, based on its semantics.

Due to the risk that reports can contain errors and that descriptions of findings can be ambiguous and difficult to interpret on account of subjective reasoning and underlying meanings (26–31), a computer-aided approach to content categorization has not been chosen for this study as it might reproduce any mistakes if they existed.

### Clinical relevance and trustworthiness

The main issues in content analysis relates to the concept of trustworthiness (18–20). Since each individual has his own pre-understanding and is prone to interpret data accordingly, there is always the risk of coding issues, alternative interpretations, or misinterpretations (17). To minimize such risks and to achieve as high an intercoder reliability and accuracy as possible in the coding process, after the initial coding was performed the coding was double checked by an abdominal radiologist and randomly selected coding was spot checked by another radiologist. Some texts were also coded more than one time to ensure coding consistency.

## Results

Our main result shows there was a gap between EBP and everyday reporting practice of the primary staging of rectal cancer. Of all reported content, 86% correlated with the EBP and the pre-defined tumor specific categories. If no findings were to be omitted according to what was being mentioned in the EBP, the total number of recording units would be at least 450 per hospital, except for one hospital with 324 recording units. Since the EBP included more information than was generally found in the reports, when adjusting for omitted information, only 48% of the reported content were accounted for. On the assumption that all categories must be used for a complete report for the primary staging of rectal cancer, this indicates 52% of clinically relevant information absent in the 2010 reports.

**Fig. 2.**
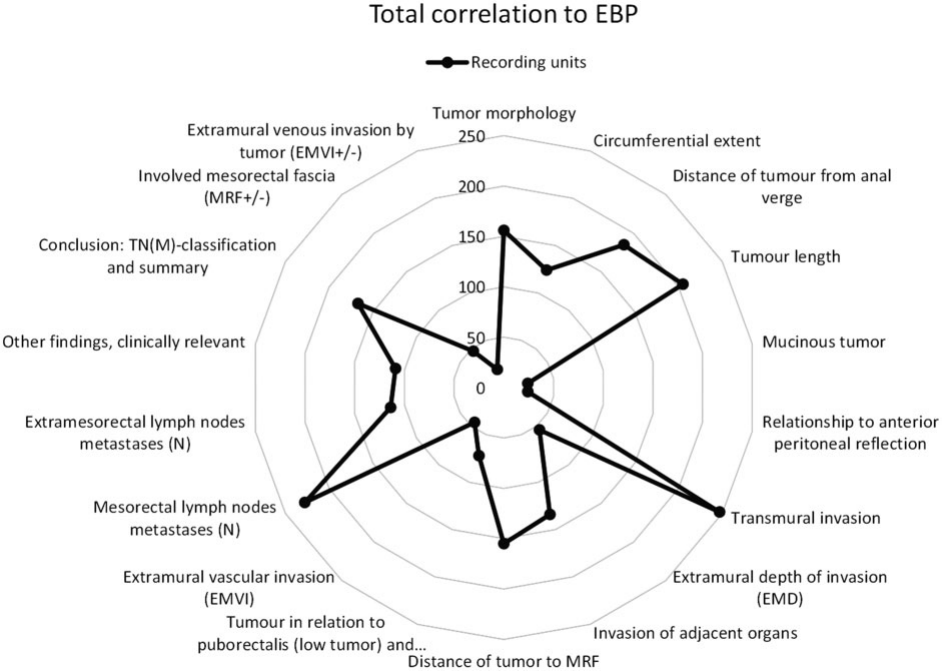
Recording units in correlation to evidence-based practice and the pre-defined tumor-specific categories. The numbers indicate how many recording units have been coded to each specific category and are an aggregate of all the reports.

**Fig. 3.**
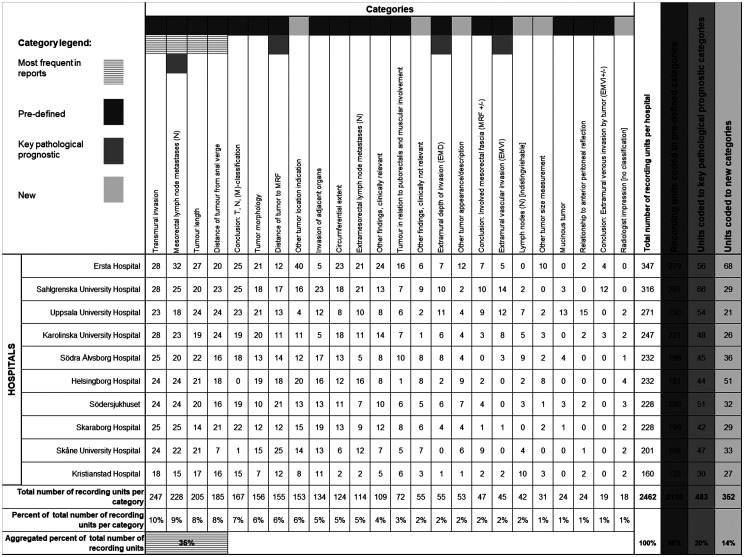
Results of coded recording units to categories. The table shows the categories with the highest count of recording units, the pre-defined categories including the categories with key findings of pathological prognostic importance. The new categories that were not part of the pre-defined coding scheme, but created during the coding process, are also presented.

The correlation to EBP and the pre-defined tumor specific categories is graphically expressed in a radar chart (Fig. 2). The overall coding result is shown (Fig. 3) with the total amount of recording units per category, hospital, and highlighted percentage in the specified areas of interest.

The most frequently used categories were categories related to describe findings of: (i) whether there is tumor invasion outside the rectal bowel wall or not (transmural invasion); (ii) local lymph node descriptions and whether these were metastases or not (mesorectal lymph node metastases); (iii) tumor length; and (iv) the distance of the lowest part of the tumor in relation to the anal verge (distance of tumor from anal verge). The recording units in these four categories accounted for 35% of the total reported content (Fig. 3).

Of the four categories considered to be of key pathological prognostic importance, the most frequently used category was the category of mesorectal lymph node metastases. Of the other three categories, the distance of tumor to mesorectal fascia had the highest count of recording units while the mentions of extramural depth of invasion and if there are any extramural vascular invasion (EMVI) were among the least used categories of all. The recording units in these four categories accounted for 20% of the total reported content.

Six categories were not part of the pre-defined coding scheme but added as new categories during the coding process (Fig. 3). All the recording units coded to one of these new categories could fit into a pre-defined theme but not a specific pre-defined category. A common denominator for these categories is that they are of the type ‘other’, i.e. they contain recording units that are somewhat alike recording units of a pre-defined category but does not fit that category. An example can be descriptions of tumor size other than length, such as thickness.

None of the new categories were among the most used. Only the category that contains other kinds of descriptions of tumor locations was being used more frequently. The other five new categories were among the least used. The recording units in the six new categories accounted for 14% of the total reported content.

## Discussion

The present study is based on a qualitative content analysis of MRI reports for the staging of rectal cancer authored in Sweden in 2010 by utilizing a pre-defined thematic coding scheme based on EBP for the staging of rectal cancer in the form of Swedish national proforma (12,13). The focus was on adherence to the EBP and: (i) what findings were being frequently most reported; (ii) to what extent the reports correlated to key pathological prognostic findings as described by the MERCURY study; and (iii) if there were clinical concepts and findings frequently reported beyond the scope of the EBP. Our main finding shows there was a significant gap between the EBP and radiology reporting in 2010 at 10 hospitals in Sweden.

Other studies have used forms of content analysis to analyze the content in electronical medical records, e.g. to compare notes to content in EMR templates (32) and to analyze free-text content of radiology reports in comparison to RSNA reporting templates (33). The method has also been used to determine semantic categories as ways of making text simplification of medical Swedish in radiology reports into general Swedish for laymen (34); however, to the best of our knowledge, no other studies have compared adherence of Swedish preoperative MRI reports to EBP for the staging of rectal cancer.

The lack of standardized IT systems and the absence of standardized detailed clinical models is a known challenge (35). Standardized IT systems with discrete data are key to data querying, data mining, and are an enormous facilitator when it comes to more sophisticated data utilization such as artificial intelligence (AI). AI research has come a long way in making computable algorithms to analyze patterns and detect pathological findings in medical images (36–38), something that requires a large amount of accurately annotated material (39). Since free-text radiology reports have been shown to be ambiguous and sometimes inaccurate (26–31), they are suboptimal for use within AI unless annotated or captured in a structured fashion. The annotation method applied in this study, using content analysis, could prove a useful tool to harmonize textual content based on the same meaning and in combination with an evidence-based reference standard there could be possibilities of implementing AI functionalities such as automatic detection of omitted information, as have been seen in this study as well as in other recent studies (40,41).

Since the participating healthcare regions and hospitals were chosen based on the reported number of rectal cancer patients to the SCRCR, where ones with higher number of patients were selected, this study covers radiology departments with more rectal cancer patients per year than the average department which could mean that the results would be different if compared to such hospitals. It can be assumed that the contents of reports in correlation to the EBP would decrease as radiologists in these hospitals are less used to the reading and reporting of rectal cancer patients.

The underlying assumption is that the categories with the higher numbers of recording units are of greater concern and importance (18). In this instance, it would mean that the frequently most reported findings are perceived to be of more importance to the reporting radiologists than findings with lower counts of recording units.

An explanation to the relatively fewer counts of recording units for some categories in comparison to other categories can be that there is no need to describe findings not present, e.g. if there is no tumor spread through the bowel wall, there is no need to also make a statement about how deep the tumor extends into the mesorectal fat, or why findings related to a low tumor are omitted if the findings relate to a tumor in the recto-sigmoidal junction. However, pertinent negatives has a value in the reports, as well as for the primary use of information in clinical process and in the automation of information in systems use such as clinical decision support (42) as in cases of secondary use, e.g. when reporting to registries, doing research, or other kind of follow-ups, which makes it important to know when and how to report such finding. This could be addressed with a standardized reporting template given the correct implementation.

The results show that the four emphasized key findings of pathological prognostic importance were not among the most frequently reported findings except for descriptions of mesorectal lymph node metastases. This category, however, is multifaceted with different types of descriptions, i.e. unspecific statements with no pathologic statements, generic descriptions of mesorectal lymph node anatomy, location, and counts, or specific statements with either clear descriptions of malignancy or that there is no sign of pathologic lymph node findings.

One could argue that the most frequently used categories, in comparison to the categories considered prognostically more important, generally requires less clinical assessment where the radiologist does not need to decide if the finding has implications for the forthgoing treatment or not. This could be an underlying factor as to why these key findings of prognostic importance have lower counts. In some cases, it could also have to do with level of expertise in reading and reporting of the staging of rectal cancer. Since there are relatively few cases per year, radiologists at smaller hospitals might lack in training material. The implementation of structured reporting would address this problem since a reporting template can act as a guide to the radiologist throughout the reading process and make sure pertinent findings are not omitted.

Recording units that could not be coded to an existing category were managed in an inductive manner and coded to a newly developed category. These new categories were generally less frequently used than the pre-defined categories and, in most cases, not more used in the reports than the categories with clinical key information. The only new category that stands out with lots of recording units is the category with descriptions of different tumor locations other than the pre-defined categories of location type. This might be a signal that there is a need to educate the standardized approach to determine the tumor location.

The initial MERCURY studies (1,3,6) and some of the following articles (4,7) were established knowledge before or around the time the reports of this study were authored. Since the coding results between content categories and variations among the different hospitals shows quite a big spread, this might signal the need of a nationally organized and orchestrated implementation of a standardized method to report findings for the staging of rectal cancer. The introduction of the national reporting template for the staging of rectal cancer (13) was introduced in 2014 by the SFMR. This might have had a positive effect on the content coverage. However, there is a question of how well and how fast new medical methods and research results become everyday practice and how new standards are being implemented.

To be able to minimize reporting differences between healthcare organizations and to ensure clinically important findings are not omitted, there is a need for a shift in how reporting is done. The challenges are to implement standardized protocols into the radiological reporting practice and the different reporting IT systems. It involves continuous collaboration with radiologists and clinicians concerning how reporting should be done regionally and nationally, as well as with the industry and the relevant standards development organizations.

In conclusion, this study illustrates there was a gap between EBP and everyday practice in 2010. Approximately half of the clinically important imaging information necessary for treatment planning described in the reference standards were omitted in the free-text reports and the emphasized key findings of pathologic prognostic importance were not among the frequently most reported findings except for findings related to the N-staging. Key findings of pathologic prognostic importance to the classification of the primary tumor, the T-stage, were not among the most commonly reported findings in everyday practice at the time these reports were authored. Several new categories were identified. However, these categories were not among the most used and seems mostly to be describing findings related to existing categories. The identified gap might be explained as a natural transition period as it takes time to implement new standards and methods.

## Appendix 1

The pre-defined coding scheme is made up of five themes, five sub-themes, and 23 categories.

The themes were created in accordance to content groupings and headings found in generic radiology practice guidelines for the communication of imaging findings in Europe (21) and North America (22).

Sub-themes were created in dialogue with a panel of abdominal radiology experts as content groupings of categories with close semantical relations within a theme, e.g. categories describing the spread of the tumor were grouped to a sub-theme relating to Tumor extension within the theme of Tumor findings.

The categories function as placeholders for the content to be analyzed, i.e. the so-called recording units (17,18,20,23–25,43), and can conceptually be divided into generic categories and rectal tumor-specific categories. The tumor-specific categories are all considered important for the primary staging report of rectal cancer; however, there are four marked key categories of pathological prognostic importance emphasized by the MERCURY studies (1,3,6). The tumor-specific categories found in the themes “Tumor findings” and “Conclusion” were retrieved from the SCRCR proforma protocol (12) and the SFMR national reporting template for the primary staging of rectal cancer (13). The four categories of generic character were extracted from the radiology practice guidelines previously mentioned. The category of clinically relevant other findings could be found in both the European practice guidelines and the SFMR reporting template.

Since the M-stage and the descriptions of distant metastases are not communicated solely based on the pelvic MRI but rather through a computed tomography (CT) scan of the thorax and abdomen (11), this category was disregarded in the results although it was mentioned in the EBP.

Hence, in the total of the 23 pre-defined categories, there were 18 categories with closer relation to the EBP of the staging of rectal cancer tumors found within the themes of “Tumor findings,” “Other findings,” and “Conclusion.”

As recommended (17), to ensure the validity of the coding scheme in total and the clinical relevance and trustworthiness of the study, a panel of abdominal radiology experts were independently asked to validate the scheme. This process was iterated until the panel reached an agreement on the labelling and the structure of the themes, sub-themes, and their relations to the categories.
